# *Mycobacterium tuberculosis* Exploits Focal Adhesion Kinase to Induce Necrotic Cell Death and Inhibit Reactive Oxygen Species Production

**DOI:** 10.3389/fimmu.2021.742370

**Published:** 2021-10-20

**Authors:** Afrakoma Afriyie-Asante, Ankita Dabla, Amy Dagenais, Stefania Berton, Robin Smyth, Jim Sun

**Affiliations:** ^1^ Department of Biochemistry, Microbiology and Immunology, University of Ottawa, Ottawa, ON, Canada; ^2^ Centre for Infection, Immunity and Inflammation, University of Ottawa, Ottawa, ON, Canada

**Keywords:** *Mycobacterium tuberculosis* (Mtb), protein tyrosine kinase 2 (*PTK2*), focal adhesion kinase (FAK), macrophage cell death, necroptosis, reactive oxygen species, host-directed therapy

## Abstract

Tuberculosis is a deadly, contagious respiratory disease that is caused by the pathogenic bacterium *Mycobacterium tuberculosis* (Mtb). Mtb is adept at manipulating and evading host immunity by hijacking alveolar macrophages, the first line of defense against inhaled pathogens, by regulating the mode and timing of host cell death. It is established that Mtb infection actively blocks apoptosis and instead induces necrotic-like modes of cell death to promote disease progression. This survival strategy shields the bacteria from destruction by the immune system and antibiotics while allowing for the spread of bacteria at opportunistic times. As such, it is critical to understand how Mtb interacts with host macrophages to manipulate the mode of cell death. Herein, we demonstrate that Mtb infection triggers a time-dependent reduction in the expression of focal adhesion kinase (FAK) in human macrophages. Using pharmacological perturbations, we show that inhibition of FAK (FAKi) triggers an increase in a necrotic form of cell death during Mtb infection. In contrast, genetic overexpression of FAK (FAK^+^) completely blocked macrophage cell death during Mtb infection. Using specific inhibitors of necrotic cell death, we show that FAK-mediated cell death during Mtb infection occurs in a RIPK1-depedent, and to a lesser extent, RIPK3-MLKL-dependent mechanism. Consistent with these findings, FAKi results in uncontrolled replication of Mtb, whereas FAK^+^ reduces the intracellular survival of Mtb in macrophages. In addition, we demonstrate that enhanced control of intracellular Mtb replication by FAK^+^ macrophages is a result of increased production of antibacterial reactive oxygen species (ROS) as inhibitors of ROS production restored Mtb burden in FAK^+^ macrophages to same levels as in wild-type cells. Collectively, our data establishes FAK as an important host protective response during Mtb infection to block necrotic cell death and induce ROS production, which are required to restrict the survival of Mtb.

## Introduction

Tuberculosis (TB) is one of the most ancient and deadliest diseases to afflict humankind, and it remains a major global health and economic threat (1). TB is a respiratory disease caused by inhaling the bacterium *Mycobacterium tuberculosis* (Mtb), which is transmitted through airborne droplets. Over the past 200 years, TB disease has been responsible for over 1 billion deaths (2), with 10 million new active TB infections and 1.5 million deaths occurring every year in recent times (3). Unfortunately, antimicrobial chemotherapy, which remains the standard choice of treatment, faces significant challenges due to the emergence of multi-drug resistant TB strains and lengthy treatment durations that are associated with poor patient compliance and high toxicity (4).

Human alveolar macrophages, the first line of defense against the inhaled bacteria (5), largely rely on multiple innate antimicrobial effector mechanisms to facilitate bacterial clearance (6, 7). Effective host control and elimination of Mtb depends on mechanisms including phagocytosis (6, 8), phagosome maturation (9), autophagy (10), antigen presentation and priming (11), metabolic pathways (12), reactive oxygen and nitrogen species production (13, 14), and programmed cell death pathways (15). However, Mtb employs a range of strategies to escape these host defences, leveraging the intracellular environment as a replication niche to promote its survival and subsequent systemic infection (16, 17).

Mtb specifically manipulates the timing and mode of cell death in infected macrophages (18, 19) to evade host immunity, thereby shielding the bacteria from destruction by the immune system and antibiotics, while allowing for the spread of bacteria when the timing is appropriate (7, 20, 21). An understanding of how this successful intracellular pathogen regulates host cell death to promote its survival is critical for developing alternative therapeutic strategies aimed at targeting host proteins/pathways such as adjunctive host-directed therapies (HDT) (22).

Cell death is a consequence of the interactions between the bacteria and the host cell (23). It can greatly influence disease progression by promoting either the survival or clearance of the invading pathogen (24–26). Mtb*-*infected macrophages can undergo two main modes of cell death: apoptosis and necrosis (27), the latter of which includes both passive and programmed forms of cell death. Apoptosis and necrosis usually have different outcomes for the course of infection. Apoptosis, a tightly regulated process of programmed cell death, minimizes inflammation by ensuring that dismembered contents of dying cells are contained within membrane-bound vesicles termed apoptotic bodies. These vesicles display “eat me” signals to facilitate recognition and elimination by neighbouring and resident phagocytes (28, 29). As such, apoptosis benefits the host by eliminating a replication niche and exposing pathogens to both cell-mediated and humoral immunity (28). Unfortunately, Mtb modulates host cell signalling molecules, including phosphatases, kinases and lipids, to control cell death to escape host immunity (17). For instance, Mtb manipulates the expression of protein phosphatase, Mg^2+^/Mn^2+^-dependent 1A (PPM1A), a key regulator of innate antibacterial and antiviral responses in macrophages, to block apoptosis (30, 31). In turn, intracellular survival of Mtb and subsequent bacterial dissemination is facilitated. Host eicosanoids, such as lipoxin A4 (LTA_4_), are targeted by Mtb to downregulate pro-apoptotic enzymes or lipids, such as cyclooxygenase 2 (COX2) and prostaglandin E2 (PGE_2_), thereby suppressing apoptosis (32). Mtb further exploits Mcl-1, a pro-survival protein, by inducing its expression to repress Bax and limit apoptosis in turn (33).

The ability of Mtb to block apoptosis suggests an alternative exit strategy allowing the pathogen to reach the extracellular environment. Mtb directs its exit from the host cell by shifting the balance from host-protective apoptotic cell death towards necrotic forms of host cell death. Necrotic cell death is characterized by loss of plasma membrane integrity, swelling of cytoplasmic organelles, and the release of cytoplasmic and nuclear contents to the extracellular space, which results in hyperinflammation (24). As such, necrotic cell death is permissive for bacterial replication and allows Mtb to disseminate to neighbouring cells, thus leading to lung damage (25, 27). In recent years, two coordinated modes of programmed necrotic cell death namely, necroptosis and pyroptosis have been described (18). Necroptosis is induced by an apoptotic stimulus under conditions where apoptosis execution is hindered whereas pyroptosis is triggered by proinflammatory signals and associated with inflammation (18). Necroptosis is usually initiated by immune ligands such as TNF-α, Fas and Lipopolysaccharide (LPS), leading to the activation of the receptor interacting serine/threonine kinase 3 (RIPK3), which phosphorylates mixed lineage kinase domain like pseudokinase (MLKL) and subsequently disturbing the cell integrity (34). On the other hand, pyroptosis requires the activation of caspase-1 which cleaves and activates inflammatory cytokines such as IL-1β and IL-18 (35). Mtb is known to induce necrosis *via* the virulence factor tuberculosis necrotizing toxin (TNT). TNT is secreted into the macrophage cytosol to degrade all cellular NAD^+^, thus leading to the activation of necroptotic cell death pathways (36, 37). Mtb has also been shown to target the p38 mitogen activated protein kinase (MAPK) signaling pathway by inducing its phosphorylation, which leads to mitochondrial membrane permeability transition and subsequent necrotic death of human macrophages (38). Necrotic cell death also plays a detrimental role in inflammatory tissue damage and may result in the loss of granuloma integrity in the lungs of infected individuals (39, 40). In summary, virulent strains of Mtb have evolved to exploit host signaling pathways to skew host cell death dynamics towards modalities that favour bacterial persistence, such as necrosis, and away from modalities that promote bacterial clearance, such as apoptosis (36, 41, 42). As such, identification and characterization of cellular checkpoints that are exploited by Mtb to modulate host cell death are key to understanding immunity against TB and could allow for the development of targeted therapeutics aimed at restoring the ability of macrophages to effectively eliminate Mtb.

Previous studies from our lab identified focal adhesion kinase (FAK) as a dysregulated signaling node in macrophages that were persistently infected with Mtb (4-6 weeks) or in macrophages that resisted apoptosis (30, 31), suggesting a potential role for this kinase in the control of cell death during Mtb infection. FAK is a cytoplasmic non-receptor protein tyrosine kinase that is encoded by the protein tyrosine kinase 2 (*PTK2*) gene (43, 44). It is located at the focal adhesion sites and is responsible for regulating cellular survival (45, 46), proliferation (47), and migration (48) in various types of cells. FAK is most well-characterized for its role in apoptosis in cancer cells (49–52) by functioning upstream of the p53 and protein kinase B (Akt)/phosphatidylinositol 3-kinase (PIP3) pathways (53). In addition, a few studies have reported a role for FAK in the regulation of diverse mechanisms necessary for the internalization or impairment of certain bacteria, viruses, and fungi into host cells (54–58).

Despite the well-characterized role of FAK in cell death in the context of cancer, its function in macrophage cell death during bacterial infection has not been well explored. Given the function of FAK in cell death and its link to persistently Mtb-infected macrophages, we hypothesized that FAK may be exploited by Mtb to modulate host cell death and promote its survival. Herein, we demonstrate that FAK expression is downregulated following Mtb infection in both human and mouse macrophage cell lines. To examine the effect of dysregulated FAK expression during Mtb infection, we used pharmacological and genetic perturbation approaches. Pharmacological inhibition of FAK increased cell death of Mtb-infected macrophages, whereas overexpression of FAK blocked cell death during infection. Mechanistically, we determined that necroptosis is the specific mode of cell death controlled by FAK during Mtb infection. Consistent with an effect of FAK modulation on cell death, inhibition of FAK resulted in enhanced replication of Mtb, whereas overexpression of FAK restricted the intracellular survival of Mtb. The ability of macrophages overexpressing FAK to limit Mtb survival was also due in part to increased production of reactive oxygen species (ROS). Overall, our findings indicate that FAK is a host-protective signaling protein required for tuberculosis immunity by restricting host macrophage necrosis. Our work provides insight into how Mtb exploits host signaling pathways to modulate the mode of cell death, which could pave the way for host-directed therapies targeting FAK. Such therapies could skew host cell death dynamics away from necrosis to limit inflammation and bacterial dissemination, thereby facilitating bacterial containment and TB treatment.

## Materials And Methods

### Cell Culture and Reagents

THP-1 Monocytes (ATCC TIB-202) and their derivatives were maintained in RPMI 1640 medium (Gibco, Gaithersburg, MD). HEK GP2-293 (Clontech-Takara, USA), RAW 264.7 (ATCC TIB-71) and mouse bone marrow-derived macrophages (BMDMs) were maintained in Dulbecco’s Modified Eagle’s Medium (DMEM) (Millipore Sigma, Burlington, MA). RPMI-1640 and DMEM media were supplemented with 2 mM L-glutamine, Penicillin-Streptomycin (100 I.U./mL penicillin, 100 µg/ml streptomycin), 10 mM HEPES and 10% heat-inactivated fetal bovine serum (FBS). Cells were maintained at 37°C in a humidified atmosphere of 5% CO_2_. BMDMs were derived from bone marrow of mouse femurs and tibiae following differentiation in DMEM supplemented with 20% L929 conditioned medium for 7-8 days. Purity of BMDM cultures was evaluated by anti F4/80 (T45-2342, BD Pharmingen, Franklin Lakes, NJ) and CD11b (M1/70) staining *via* flow cytometry. THP-1 monocytes were differentiated with 100 ng/ml Phorbol 12-myristate 13-acetate (PMA, Alfa Aesar, Haverhill, MA) for 72 h, after which the differentiated cells were washed with Phosphate buffered Saline (PBS) prior to infection with Mtb.

### Inhibitors

PF-573-228 and necrostatin-1 were purchased from MedChem Express (Monmouth, NJ), while necrostatin-1s was purchased from New England Biolabs (Ipswich, MA). GSK-872 was purchased from Tocris (Toronto, Canada). Necrosulfonamide (NSA), N-acetyl cysteine (NAC), PF-562-271, and GSK2795039 were purchased from Millipore Sigma.

### Bacteria and Plasmids

The Mtb H37Rv-derived auxotrophic strain mc^2^6206 (Δ*PanCD*Δ*LeuCD*) (59) was grown in Middlebrook 7H9 medium supplemented with 0.2% glycerol (Fisher Chemical, Waltham), 0.05% Tween-80 (Acros Organics, Fair Lawn, NI), and 10% OADC and maintained at 37°C with slow shaking (50 rpm). Growth medium of the auxotrophic Mtb strain was supplemented with 24 μg/ml pantothenate and 50 μg/ml L-leucine. Mtb mc^2^6206 expressing firefly luciferase was generated by transformation of the pSMT3-luciferase plasmid (60). Mtb-luciferase were maintained in selection with 50 µg/ml Hygromycin B (Calbiochem, San Diego, CA). Mtb mc^2^6206 expressing tdTomato was generated by transformation of the pML2304 plasmid (generous gift from Dr. Michael Niederweis) and maintained in selection with 50 µg/ml Hygromycin B. *Escherichia coli* strains DH5α and NEB Stable (New England Biolabs) were used for plasmid propagation production of recombinant FAK protein and were grown in Luria-Bertani broth at 37°C. Plasmid pMSCV-FAK was generated using the Gibson assembly method with oligonucleotides designed using the NEBuilder Assembly Tool. The FAK gene (*PTK2*) was amplified in two fragments from THP-1 cell-derived cDNA. The first fragment was amplified using the oligonucleotide pair 5’-aggcgccggaattagatctcATGGCAGCTGCTTACCTTG-3’ (forward; lowercase letters denote overlap with vector backbone, uppercase letters denote gene-specific primer) and 5’-aaccgccaaaGCTGGATTCTCTGGACTC-3’ (reverse; lowercase letters denote overlap with 2nd FAK fragment, uppercase letters denote gene-specific primer). The second fragment was amplified using the oligonucleotide pair 5’-agaatccagcTTTGGCGGTTGCAATTAAAAC-3’ (forward; lowercase letters denote overlap with 1st FAK fragment, uppercase letters denote gene-specific primer) and 5’-cctacccggtagaattcgttTCAGTGTGGTCTCGTCTG-3’ (reverse; lowercase letters denote overlap with vector backbone, uppercase letters denote gene-specific primer). pMSCV-puro was digested using the restriction enzymes XhoI and HpaI, and the two PCR amplified FAK fragments were inserted into the multiple cloning site for retroviral expression.

### Generation of THP-FAK^+^ Cells

HEK GP2-293 cells seeded at 50% confluency were co-transfected with retroviral vector pMSCV-FAK and the VSV-G envelope plasmid using FuGENE (Promega, Madison, WA) at a ratio of 4:1 (FuGENE : DNA). Culture supernatants were harvested after 48 h, aliquoted, and stored at -80°C. Different volumes of supernatants containing retroviral particles supplemented with 10 µg/ml DEAE-Dextran (Sigma-Aldrich, UK) were used to transduce THP-1 cells, after which the viability of transduced cells was examined. Cells were selected by puromycin and analyzed by western blot to verify overexpression of FAK protein.

### Bacterial Infection and Survival

Mtb mc^2^6206 growing in log-phase was quantified by optical density measurement at 600 nm (OD_600_) using the conversion of 3 × 10^8^ (Mtb) bacteria per ml for an OD_600_ of 1.0. The number of bacteria required for various multiplicity of infections (MOIs) were washed and resuspended in RPMI 1640 media without antibiotics supplemented with 10% human serum. For infection of differentiated THP-1 macrophages, RAW 264.7 macrophages, and BMDMs, bacteria were added to the cells and incubated at 37°C for 4 h. Cells were then washed 3 times with PBS to remove extracellular, non-phagocytosed bacteria and the infection was continued at 37°C for the desired time. For infections performed in the presence of the FAK inhibitors PF-573-228 or PF-562-271, cells were pre-treated 24 h before infection. Compounds were maintained in the medium for the duration of the experiment and refreshed every second day. To assess the survival and viability of intracellular Mtb, cell culture supernatant was removed, and Mtb-infected cells were washed three times with PBS. Macrophages were lysed with Glo Lysis Buffer as per the manufacturer’s instructions at the indicated time points post-infection. Luciferase activity, proportional to the number of viable bacteria in each well, was determined using the Bright-Glo™ Luciferase Assay System according to the manufacturer’s protocol. Resultant luminescence was measured with the Synergy™ H1 Hybrid Multi-Mode Reader (BioTek, Winooski, Vermont) using 96-well solid white plates (Corning, Corning, NY) and an integration time of 1 second per well.

### Resazurin Cell Viability Assay

The resazurin microtitre plate assay was used to determine the cytotoxicity of FAK inhibitor compounds. At the indicated time points, resazurin working solution was added to each well to a final concentration of 0.03 mg/ml. After a 2 h incubation at 37°C, fluorescence was measured using the Synergy™ H1 Hybrid Multi-Mode Reader with excitation wavelength of 560 nm and emission wavelength of 590 nm. Cytotoxicity of cells was assessed by comparing the fluorescence of treated to untreated cells. To assess the antibacterial effects of FAK inhibitors, serial dilutions of compounds were added to each well containing 1.2 x 10^7^ Mtb. The culture plate was sealed with parafilm and incubated at 37°C for 5 days. Then, resazurin solution was added to each well to a final concentration of 0.03 mg/ml. After a 6 h incubation at 37°C, fluorescence was measured as indicated above.

### Real-Time Cell Analysis Assay (RTCA)

Macrophage adhesion was measured in specialized 96-well plates (E-plate 96) with the xCELLigence Real-time Cell Analyzer (RTCA) SP apparatus (ACEA Biosciences, San Diego, CA). Data was quantified by measuring impedance changes between the sensing electrodes located in the well-bottom, which changes as a function of the adhesion of cells to the surface of the plate. A dimensionless value, the Cell Index (CI) is representative of these impedance changes. Using this system, macrophage adhesion—and therefore viability—was monitored in real-time. Plates were removed after 72 h of THP-1 macrophage differentiation for the addition of Mtb for infection. After each manipulation, plates were placed back in the RTCA apparatus for kinetic monitoring. The CI at every time point represents the mean of three biological replicates.

### FVS 780 Live/Dead Assay

Plasma membrane permeability, an indicator of necrotic cell death, was determined using the BD Horizon™ Fixable Viability Stain 780 (FVS780) (BD Biosciences). Cell culture supernatants were collected to preserve detached cells. Cells were washed twice with PBS and TrypLE Express enzyme was added to wells. The plate was incubated at 37°C for 5 min to allow for thorough detachment of adherent cells. Harvested cells were washed twice with PBS and stained with BD Horizon™ Fixable Viability Stain 780 according to the manufacturer’s protocol. Flow cytometry analysis was then performed to quantify viable or dead cells.

### Cell Titer-Glo Assay

Metabolic activity, proportional to the number of viable cells in each well, was determined using the Cell Titer-Glo Assay System according to the manufacturer’s protocol (Promega). At the indicated time points post-infection, equal volumes of Cell Titer-Glo reagent was added to the volume of cell culture supernatant present in each well. Contents were mixed for 2 min on an orbital shaker to induce cell lysis. Plates were then incubated for 10 min to stabilize luminescence signal. Resultant luminescence was measured with the Synergy™ H1 Hybrid Multi-Mode Reader using 96-well solid white plates and an integration time of 1 second per well.

### Annexin V Assay

Cell culture supernatants were collected to preserve detached cells. Cells were washed with twice with PBS and TrypLE Express enzyme was added to the wells. The plate was incubated at 37°C for 5 min to allow for through detachment of adherent cells. Harvested cells were washed twice with PBS and stained with Annexin V conjugated to FITC (e-Biosciences, San Diego, CA) in combination with Fixable Viability Stain 780 according to the manufacturer’s protocol. Flow cytometric analysis was then used to quantify the amount of healthy (Annexin V-, FVS780-), apoptotic (Annexin V+, FVS780-), and necrotic cells (Annexin V-, FVS780+ and Annexin V+, FVS780+).

### Lactate Dehydrogenase Assay 

The release of lactate dehydrogenase in culture supernatants, an indicator of necrotic cell death, was quantified using the CYQUANT LDH cytotoxicity detection kit (Thermo Fisher Scientific, Waltham, MA) according to the manufacturer’s instructions. The assay was read using a Synergy H1 microplate reader at 490 nm with a wavelength correction at 680 nm.

### Flow Cytometry

Select cell viability assays were analyzed by flow cytometric analysis using the CYTOFLEX (Beckman Coulter, Indianapolis, IN). Data analysis was performed using CytExpert software (Beckman Coulter) or FlowJo V10 software (BD Life Sciences, Ashland, OR). Flow cytometry was conducted to measure cell density and viability using scattering properties. 

### Cytokine Profiling

Cytokine expression was measured in culture supernatants harvested from control and Mtb-infected THP-1 macrophages at 24 h post-infection. The Human ELISA kit (Invitrogen, Carlsbad, CA) was used according to the manufacturer’s instructions to measure the expression of TNF-α, IL-1β and IL-10. All samples were read using the Synergy™ H1 Hybrid Multi-Mode Reader at 450 nm.

### Quantitative Real-Time PCR

RNA was isolated from THP-1 or RAW 264.7 macrophages using the Aurum Total RNA Mini Kit (Bio-Rad, Hercules, CA). cDNA was synthesized from 500 ng of total RNA. Gene expression of *PTK2* or the reference gene was analysed by real-time PCR using primers designed as per MIQE guidelines (61) and Sso Advanced SYBR Green Supermix (Biorad) in a CFX96 Touch Real-Time PCR Detection system. The 2^(-ΔΔCt)^ method (62) was used to determine relative gene expression as normalized to *ACTB*, and relative fold expression changes was normalized to uninfected macrophages (day 0) as 1.0. Oligonucleotide sequences used were as follows: human *PTK2*, 5’-TACAACGAGGGTGTCAAGCC-3’ and 5’- GCCCGTCACATTCTCGTACA-3’; mouse *Ptk2*, 5’-CGGACACATGCAGTCTCTGT-3’ and 5’-CGAGGGCATGGTGTATGTGT-3’; human *ACTB*, 5’-ATTGCCGACAGGATGCAGAA-3’ and 5’-GCTGATCCACATCTGCTGGAA-3’; mouse *GAPDH*, 5′-GGCAAATTCAACGGCACAGT-3′ and 5′-AGATGGTGATGGGCTTCCC-3′.

### Immunoblot

Macrophages were harvested by centrifugation, washed once with PBS, and lysed in RIPA buffer (150 mM NaCl, 1.0% IGEPAL^®^ CA-630, 0.5% sodium deoxycholate, 0.1% SDS, 50 mM Tris, pH 8.0) containing protease and phosphatase inhibitors (Halt™ Protease and Phosphatase Inhibitor Cocktail, Thermo Fisher Scientific, Waltham, MA) according to the manufacturer’s instructions. Protein concentration of the lysates was determined using the RC DC™ Protein Assay kit (Bio-Rad) according to the manufacturer’s protocol. About 20 μg of protein per sample was separated *via* SDS-PAGE using hand cast gels (SureCast Gel Handcast System, Invitrogen, Carlsbad, CA) or pre-cast 4-20% gels (Mini-PROTEAN^®^ TGX Gel, Bio-Rad) and subsequently transferred to a polyvinylidene difluoride (PVDF) membrane using a wet transfer system (Mini-PROTEAN^®^ Tetra Vertical Electrophoresis Cell, BioRad). Western blot analysis was performed according to standard protocols. The Caspase-3 Control Cell Extracts (#9663) and the primary rabbit antibodies to FAK (#13009S), cleaved caspase 3 (#9661S), and total caspase 3 (#9665) were purchased from Cell Signalling Technology (Danvers, MA). Primary monoclonal mouse antibodies to GAPDH (GA1R) and Beta-tubulin (BT7R) were purchased from Invitrogen (Carlsbad, USA). A horseradish peroxidase-conjugated goat anti-rabbit or goat anti-mouse polyclonal antibody (Bio-Rad) was used as the secondary antibody. Membranes were developed using the Clarity Western Enhanced Chemiluminescence Western Substrate (Bio-Rad, Hercules, CA). Imaging and densitometry analysis were performed with Image Quant LAS4000 and software (GE Healthcare Life Sciences, Cytiva).

### Reactive Oxygen Species (ROS) Assay

Intracellular ROS was measured by labelling cells with 2’-7’Dichlorofluorescin diacetate DCFH-DA (Sigma Aldrich, UK) for 30 min at 37°C at the indicated time points post-infection. Stained cells were washed twice with PBS and fluorescence was measured at a wavelength of 485 nm/535 nm. Fluorescent images were taken using ZOE Fluorescent Cell Imager (Bio-Rad).

### Statistical Analysis

**Figure 1:**
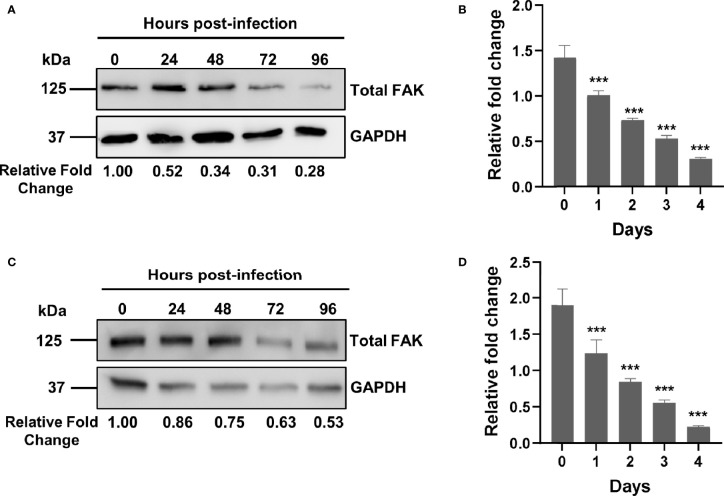
Mtb infection downregulates FAK expression. **(A)** THP-1 macrophages were infected with Mtb at a multiplicity of infection (MOI) of 10. Cell lysates were prepared at the indicated time points post-infection, and total FAK protein levels were analyzed by western blot. **(B)** *PTK2* expression levels in THP-1 macrophages infected with Mtb as in **(A)** were quantified using qRT-PCR at the indicated days post infection. **(C)** RAW 264.7 cells were infected Mtb as in **(A)**, and cell lysates were prepared at the indicated time post infection. Blots shown in **(A, B)** are representative of three independent experiments. Densitometry analysis of the blots was performed using Image Quant LAS 4000 software with intensities normalized to GAPDH. Relative fold change of total FAK levels was expressed relative to uninfected cells. **(D)** *Ptk2* expression levels in RAW 264.7 macrophages were measured as in **(B)**. qRT-PCR data were analysed using the ΔΔCT method normalizing to **(B)** *ACTB* or **(D)** *GAPDH* as a reference gene, and fold change is expressed relative to uninfected cells. qRT-PCR data represent the mean of three replicates. ***p < 0.001.

Data are expressed as the mean ± the standard deviation of three independent biological replicates. Statistical analysis was performed using two-way Anova in all figures except for **Figures 1B, D**, where one-way Anova was performed. Values of *p* < 0.05 were considered significant (*p < 0.05, **p < 0.01, ***p <0.001, *ns*: non-significant). All statistical analyses were performed using Graph Pad Prism Version 9 (Graph Pad Prism Software, San Diego, CA).

## Results

### Mtb Downregulates FAK in Macrophages During Infection

While a role for FAK has been reported in the control of different bacterial infections such as *Escherichia coli and Staphylococcus aureus* (54, 55), there are no studies examining its role in the innate immune response to Mtb infection. Using an antibody-based proteomic approach, we had previously found that FAK is dysregulated in persistently Mtb-infected macrophages and in macrophages engineered to overexpress a pro-survival host phosphatase PPM1A, which plays a key role in the antibacterial response to Mtb infection (30, 31). As such, we first sought to examine whether Mtb infection alters the expression of FAK, with the subsequent goal of determining whether FAK plays a role in the control of host cell death during Mtb infection. To address these questions, we primarily used the human THP-1 monocyte/macrophage cell line, as it is widely used to study the cellular immune response to Mtb infection (63) due to immunological and functional similarities that mimic properties of primary human macrophages (64). For infection experiments, the Mtb H37Rv derived auxotroph strain mc^2^6206 was used due to its advantage in being approved for use in biosafety level (BSL) 2 facilities. Importantly, Mtb mc^2^6206 retains all virulence factors produced by virulent strains and possesses similar *in vitro* and intra-macrophage replication rates, similar responses to TB antibiotics, and whole genome sequence conservation when compared to its parent H37Rv strain (65). Total levels of FAK protein were assessed by western blot analysis in THP-1 macrophages that were infected with Mtb for a period of 4 days. By 24 hours post-infection, total FAK protein levels were two-fold lower compared to uninfected macrophages. FAK protein expression continued to decline with the length of infection, reaching a level of ~25% relative to wild type control cells (**Figure 1A**). We further determined that the decrease in FAK protein levels during Mtb infection occurs at the level of transcription as qRT-PCR analysis showed that mRNA transcript levels of *PTK2*, the gene encoding for FAK, decreased in a similar manner over the course of infection (**Figure 1B**). The time-dependent reduction in FAK protein as well as mRNA expression levels was recapitulated in Mtb-infected RAW 264.7 cells (**Figures 1C, D**), a murine macrophage cell line routinely used in Mtb studies (66). Our data show that Mtb disrupts FAK expression early after infection, suggesting a role for this kinase in host immunity to TB.

### Modulation of FAK by Pharmacological Inhibition and Genetic Overexpression in Human and Mouse Macrophages 

**Figure 2:**
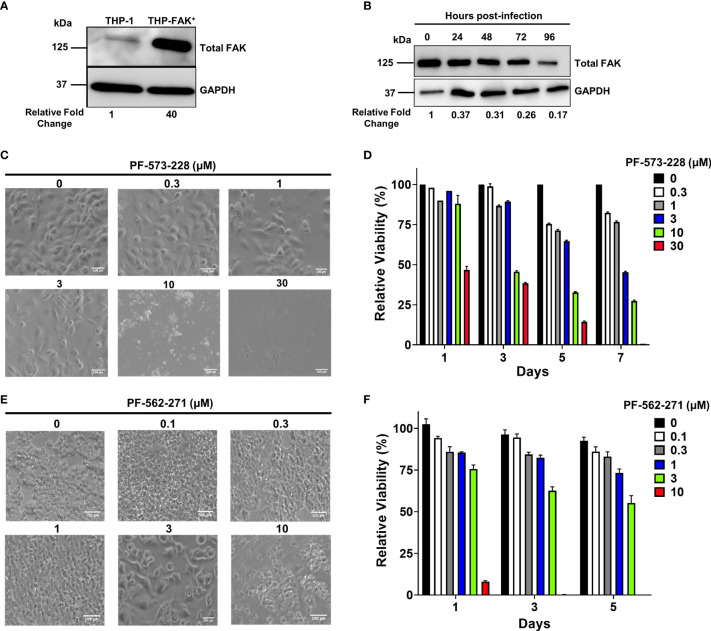
Genetic overexpression and pharmacological inhibition of FAK. **(A)** Total FAK protein levels in cell lysates of THP-1 and THP-1 cells stably overexpressing FAK (THP-FAK^+^) were analyzed by western blot. **(B)** THP-FAK^+^ macrophages were infected with Mtb at an MOI of 10 and cell lysates were prepared at indicated time points, and analysed by western blot. Blots shown in **(A, B)** are representative of three independent experiments. Densitometry analysis of the blots were performed using Image Quant LAS 4000 software with intensities normalized to GAPDH. Relative fold change of total FAK levels was expressed relative to THP-1 cells **(A)** and uninfected THP-FAK^+^ cells **(B)**. **(C, D)** THP-1 macrophages were treated with varying concentrations of PF-573-228 for 5 days, and **(C)** representative phase-contrast microscopy (20x objective) images are shown along with **(D)** quantification of cell viability using the resazurin assay. **(E, F)** RAW 264.7 macrophages were treated with varying concentrations of PF-562-271 for 5 days, and **(E)** representative phase-contrast microscopy (20x objective) images are shown along with **(F)** quantification of cell viability using the resazurin assay. Images are representative of 3 visual fields for each inhibitor concentration. Relative viability is normalized to untreated control cells as 100%. Error bars represent the mean ± SD of three independent biological replicates. Scale bar, 100 μm.

Given that FAK protein levels are downregulated during Mtb infection, we sought to address whether modulation of FAK affects the antibacterial response of macrophages. To study the role of FAK in the macrophage antibacterial response, we first attempted to genetically modify THP-1 cells to overexpress or delete FAK. We successfully overexpressed FAK in THP-1 cells using a retroviral expression vector encoding the *PTK2* gene. The resulting FAK cell line (THP-FAK^+^) produced 40-fold higher expression of FAK compared to control THP-1 cells (**Figure 2A**). Given the high levels of ectopically expressed FAK, we questioned whether Mtb infection of the FAK overexpressing macrophages would still result in detectable downregulation of FAK. Like wild-type macrophages, FAK protein levels were indeed reduced to 17% - 37% over a 4-day infection period relative to uninfected FAK^+^ macrophages (**Figure 2B**). The magnitude of decrease in FAK protein levels indicated that Mtb infection can modulate ectopically expressed FAK even though it is under the control of a constitutive promoter. While the mechanism for this remains unclear, FAK^+^ cells still produce higher levels of FAK compared to wild-type macrophages during Mtb infection; however, FAK protein levels in FAK^+^ macrophage during Mtb infection appear physiologically relevant. Attempts to knockout FAK in THP-1 cells using CRISPR/Cas9 genome-editing were unsuccessful despite using six different single guide RNA sequences that targeted different regions of the early exons in the *PTK2* gene. The inability to produce a full knockout of FAK may be consistent with earlier reports that showed whole-body deletions of FAK in mice resulted in embryonic lethality, suggesting an essential role for FAK in cells during development (67).

To overcome this problem, we opted for an alternative approach by using pharmacological inhibitors of FAK. PF-573-228, produced by Pfizer Inc (68)., was selected for our study as it is the most successful FAK inhibitor used for *in vitro* studies (69). PF-573-228 is a selective, ATP-competitive inhibitor that prevents FAK autophosphorylation at tyrosine 397 (Y397) (69). PF-573-228 is the parent compound of FAK-directed drugs (VS-6062) that are currently under clinical evaluation by Verastem. VS-6062 has completed the phase 1 clinical trial in which the primary objective was to determine safety, pharmacokinetics, and pharmacodynamics in patients with advanced malignancies (68). Given that compounds can present cytotoxicity and non-specific effects, it was important to first titrate the compounds to assess cytotoxicity and to select an optimal concentration that minimizes non-specific effects. THP-1 macrophages were treated with various concentrations of the FAK inhibitor, PF-573-228 for 5 days. Macrophage morphology and metabolic activity were then evaluated using microscopy and resazurin cell viability assays, respectively. The morphology of THP-1 macrophages was not affected by FAK inhibitor treatment at concentrations up to 3 µM, since macrophages exhibited the flattened, pancake-like morphology indicative of healthy macrophages (**Figure 2C**). However, THP-1 macrophages were more sensitive to higher doses of FAK inhibitor treatment (10 µM and 30 µM), which resulted in extensive cell death with features such as cell shrinkage, detachment, and formation of apoptotic bodies (**Figure 2C**). Consistent with the microscopy, resazaurin viability assays demonstrated that there were no differences in the viability of macrophages treated with concentrations of FAK inhibitor ranging from 0.3-3 μM (**Figure 2D**). However, PF-573-228 was highly cytotoxic at doses higher than 3 μM, which significantly lowered the viability of macrophages (**Figure 2D**). These findings indicate that treatment with 3 μM or less of PF-573-228 does not detrimentally affect THP-1 macrophage morphology or viability. Our data are consistent with a previous study showing that PF-573-228 inhibits FAK kinase activity at concentrations between 0.3 μM to 30 μM in human macrophages and cancer cells (69), and as such, 3 μM was selected as the optimal dose of PF-573-228 for human cell culture-based experiments. Given that FAK expression in Mtb-infected mouse macrophages mirrors that of human THP-1 macrophages (**Figures 1A–D**), we also searched for a suitable FAK inhibitor for mouse cells, which resulted in the selection of PF-562-271 (70). PF-562-271 is a methane sulphonamide diaminopyrimidine developed by Pfizer (68). A second generation of this inhibitor named VS-6063 (defactinib) is currently in phase I and II clinical trials for the treatment of various cancers in combination with immunotherapies and other small molecule inhibitors (71). The potential cytotoxicity of the mouse-specific FAK inhibitor PF-562-271 was assessed on RAW 264.7 cells (**Figure 2E**). Morphology of RAW 264.7 cells was not affected at concentrations up to 1 μM (**Figure 2E**). However, evidence of hypertrophy and cytotoxicity was observed at concentrations of 3 and 10 μM, as indicated by enlarged and stretched cells, and excessive cell shrinkage and debris formation, respectively (**Figure 2E**). Consistent with these findings, the viability of PF-562-271-treated RAW 264.7 cells remained comparable to control cells up to a concentration of 1 µM, which was chosen as the optimal dose of FAK inhibitor for subsequent murine cell culture-based assays (**Figure 2F**).

### Modulation of FAK Alters the Immunological Profile of Macrophages During Mtb Infection

**Figure 3:**
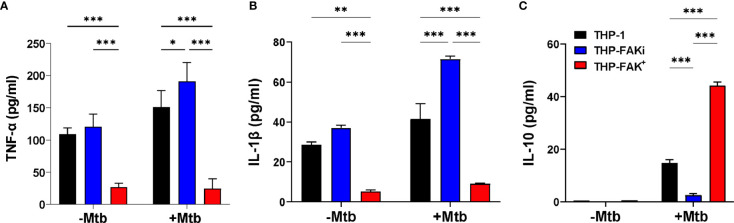
FAK expression alters cytokine production by macrophages infected with Mtb. THP-1, THP-FAKi and THP-FAK^+^ macrophages were infected with Mtb at an MOI of 10 and cell culture supernatants were collected at 24 hours post-infection. Production of **(A)** TNF-α **(B)** IL-1β **(C)** IL-10 were measured in cell culture supernatants using a human ELISA assay kit. Error bars represent the mean ± SD of three independent biological replicates. *p < 0.05, **p < 0.01, ***p < 0.001.

To determine if FAK regulates the macrophage antibacterial response to Mtb infection, we examined the effect of FAK expression on cytokine production. THP-1, THP-FAKi (THP-1 treated with FAK inhibitor) and THP-FAK^+^ (THP-1 overexpressing FAK) macrophages were infected with Mtb and culture supernatants were collected at 24 hours post-infection to quantify the levels of TNF-α, IL-1β and IL-10 using the solid-phase sandwich ELISA. Baseline (uninfected) levels of proinflammatory cytokines, TNF-α and IL-1β were observed in culture supernatants of THP-1 and THP-FAKi macrophages, but were significantly lower in THP-FAK^+^ macrophages (**Figures 3A, B**). Inhibition of FAK in THP-1 macrophages increased production of TNF-α (**Figure 3A**) and IL-1β (**Figure 3B**) upon infection with Mtb compared to control THP-1 macrophages, whereas FAK overexpression blocked the production of TNF-α (**Figure 3A**) and IL-1β (**Figure 3B**). In contrast, baseline (uninfected) levels of IL-10 were below the limit of detection in all three cell lines and induced upon infection. While FAK inhibition significantly reduced IL-10 levels, overexpression of FAK increased IL-10 production in Mtb-infected macrophages (**Figure 3C**). Although TNF-α and IL-1β are crucial for the inflammatory response against Mtb, excess levels are linked to macrophage cell death, thereby promoting hyperinflammation, persistent lung damage, and subsequent failure to clear the bacteria (6). While certain anti-inflammatory mediators such as IL-10 could pose detrimental roles during TB pathogenesis, they could also be beneficial depending on the timing of their deployment (72). Hence, our data suggest increased inflammation associated with FAK inhibition and decreased inflammation with increasing levels of FAK. These observations support a role for FAK in the inflammatory response to Mtb infection and possibly a function in the regulation of macrophage cell death.

### FAK Blocks Mtb-Induced Cell Death in Macrophages

**Figure 4:**
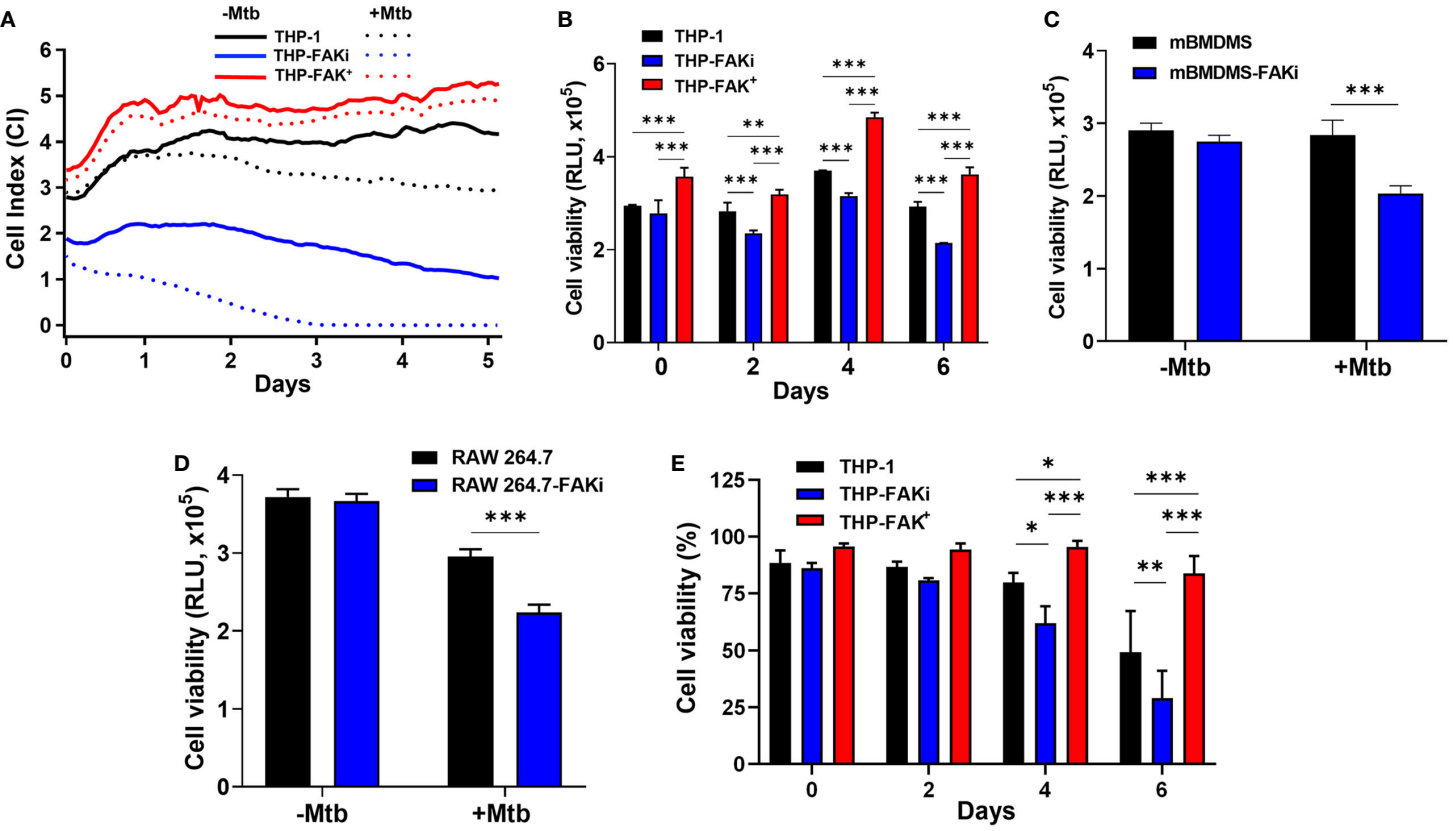
FAK is required to block cell death of Mtb-infected macrophages. **(A)** THP-1, THP-FAKi and THP-FAK^+^ macrophages were infected with Mtb at an MOI of 10, after which changes in adherence (viability) were monitored by RTCA as indicated by the Cell Index (CI). CI was measured continuously every hour for 120 hours post-infection. The data represent the mean of three independent biological replicates. **(B)** THP-1, THP-FAKi, THP-FAK^+^ macrophages, **(C)** mBMDMs, and **(D)** RAW 264.7 cells were infected with Mtb at an MOI of 10 and cell viability was quantified using Cell Titer-Glo (measured as Relative Luminescence Units, RLU). Data in **(C, D)** represent 4 days post-infection. **(E)** THP-1, THP-FAKi, and THP-FAK^+^ macrophages were infected with Mtb at an MOI of 10, and at the indicated time points post-infection, cells were stained with Fixable Viability Stain (FVS780) dye and analyzed using flow cytometry to measure the percentage of viable cells. Error bars represent the mean ± SD of three independent biological replicates. *p < 0.05, **p < 0.01, ***p < 0.001.

Our cytokine profile data provided valuable insight into the possible function of FAK in the control of macrophage cell death, and we hypothesized that Mtb exploits FAK to control the mode of host cell death. To determine the role of FAK on macrophage cell death, we used the xCELLigence Real-Time Cell Analysis (RTCA) instrument, which quantifies the viability of adherent cells over time (73). The RTCA system allows for label-free and dynamic monitoring of cellular phenotypic changes in real-time using impedance as a readout. Changes in impedance between electrodes at the bottom of E-well plates are then translated into a Cell Index (CI) value, providing quantitative information about the biological status of the cells. An increase in CI reflects an increase in macrophage adherence or viability, whereas its decrease reflects a loss of macrophage viability as they detach from the bottom of the well (30). THP-1, THP-FAKi, and THP-FAK^+^ macrophages were infected with Mtb and macrophage viability was monitored using RTCA over a period of 5 days post-infection. RTCA analysis showed that inhibition of FAK triggered increased cell death in Mtb-infected macrophages compared to control THP-1 macrophages, while overexpression of FAK completely blocked macrophage cell death (**Figure 4A**). These findings highlight a role of FAK expression in the control of macrophage cell death during Mtb infection. Results from the RTCA assay were confirmed using the Cell Titer-Glo assay, which measures metabolic activity of macrophages based on the levels of ATP present in the cells. The Cell Titer-Glo assay measures relative levels of cell death as indicated by a dose-dependent loss in luminescence signal from macrophages in response to treatment with increasing concentrations of sanguinarine, a cytotoxic compound (74) (**Figure S1**). Consistent with the RTCA data, inhibition of FAK significantly decreased the metabolic activity of Mtb-infected macrophages by 23% compared to control macrophages at day 6 post-infection (**Figure 4B**). In addition, Mtb-infected THP-FAK^+^ macrophages showed a 24% and 30% increase in ATP content compared to control macrophages at days 4 and 6 post-infection, respectively (**Figure 4B**). Indeed, inhibition of FAK in both primary BMDMs (**Figure 4C**) and RAW 264.7 macrophages (**Figure 4D**) resulted in decreased cell viability during Mtb infection compared to untreated control macrophages. Collectively, our data suggest that FAK functions to restrict host macrophage cell death during Mtb infection, which is consistent with the reduced production of TNF-α and IL-1β in macrophages overexpressing FAK.

However, since the Cell Titer-Glo viability assay does not provide information on the mode of cell death, we turned to the amine-binding live/dead cell viability assay (FSV780) to examine whether plasma membrane damage occurred alongside the cell death observed in **Figures 4A–D**. THP-1, THP-FAKi, and THP-FAK^+^ macrophages were infected with Mtb over a 6-day period, and cell viability was assessed using FSV780. Consistent with results from the other two cell viability assays (**Figures 4A–D**), FSV780 staining showed that inhibition of FAK significantly decreased the viability of Mtb-infected macrophages at days 4 and 6 post-infection, whereas increased FAK expression significantly reduced macrophage cell death (**Figure 4E**). These results demonstrate the presence of plasma membrane damage during Mtb-induced cell death, which suggests that FAK may regulate a necrotic form of cell death during Mtb infection. Given that necrotic cell death is usually associated with increased inflammation, such a hypothesis would be consistent with the increased production of inflammatory cytokines observed when FAK is inhibited (**Figure 3**).

### FAK Mediates a Necroptotic Form of Cell Death During Mtb Infection

**Figure 5:**
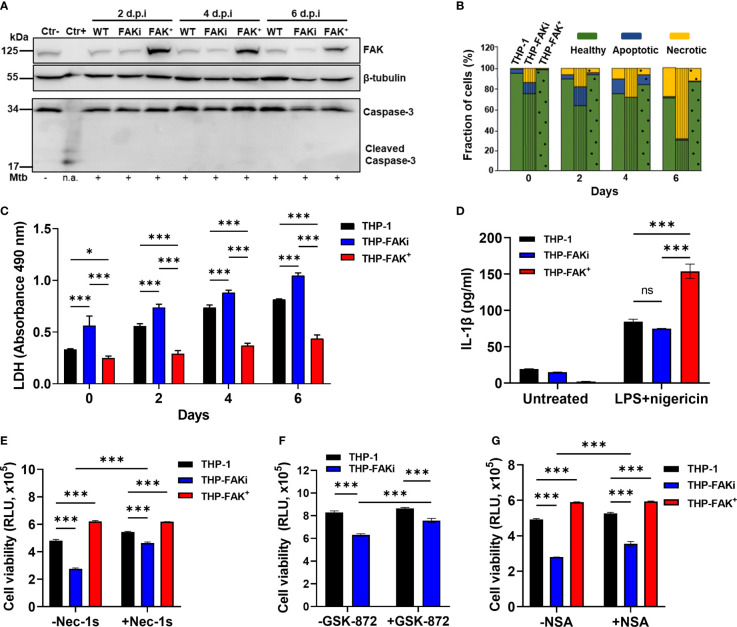
FAK controls necroptotic cell death in macrophages during Mtb infection. **(A)** THP-1, THP-FAKi, and THP-FAK^+^ macrophages were infected with Mtb at an MOI of 10, and cell lysates were prepared at the indicated days post-infection (d.p.i.). Total and cleaved caspase 3, and total FAK protein levels were analyzed by western blotting. Non-infected THP-1 cell lysates were used as a negative control for caspase 3 activation, while lysates containing cleaved caspase-3 (#9663, Cell Signaling Technologies) were used as a positive control. β-tubulin was used as loading control. **(B)** THP-1, THP-FAKi, and THP-FAK^+^ macrophages were infected as in **(A)** and at the indicated days post-infection, cells were stained with annexin V and FVS780. Stained cells were analyzed by flow cytometry to quantify the percentage of healthy, apoptotic, and necrotic cells as described in the Methods. **(C)** THP-1, THP-FAKi, and THP-FAK^+^ macrophages were infected with Mtb at an MOI of 10, and lactate dehydrogenase (LDH) released in culture supernatants were assessed using the CYQUANT LDH kit at indicated days post-infection. The amount of LDH in the supernatant is proportional to the measured absorbance at 490 nm. **(D)** THP-1, THP-FAKi and THP-FAK^+^ macrophages were mock treated or pre-treated with 1 µg/ml LPS for 4 h, followed by treatment with 5 µM nigericin for 24 h Macrophage viability was assessed using the Cell Titre-Glo assay. **(E–G)** THP-1, THP-FAKi, and/or THP-FAK^+^ macrophages were mock treated or pre-treated with **(E)** necrostatin-1s (Nec-1s, 10 µM), **(F)** GSK-872 (5 µM), or **(G)** necrosulfonamide (NSA, 10 µM) for 24 h Cells were then infected with Mtb at an MOI of 10 for 6 days, and cell viability was assessed using the Cell Titer-Glo assay. Error bars represent the mean ± SD of three independent biological replicates. *p < 0.05, ***p < 0.001, *ns*, non-significant.

To determine the specific mode of cell death controlled by the dysregulation of FAK during Mtb infection, we first assessed apoptosis since FAK is best characterized for its control of apoptosis in cancer (49). Using western blotting, we evaluated the expression of cleaved caspase 3, a reliable marker for apoptosis, in Mtb-infected THP-1, THP-FAKi, and THP-FAK^+^ macrophages for up to 6 days post-infection. No cleaved caspase 3 could be detected in uninfected or Mtb-infected THP-1 cells, which is consistent with the established paradigm that Mtb actively blocks apoptosis (**Figures 5A** and **S2**). Importantly, despite increasing cell death of infected macrophages (**Figure 4**), inhibition of FAK did not lead to any detectable cleaved caspase 3 (**Figure 5A**). Western blot results were confirmed using an Annexin V binding assay, which measures early apoptotic events in combination with a plasma membrane permeability dye that excludes necrotic forms of cell death. We observed low and comparable levels of Annexin V-positive cells that also maintained an intact plasma membrane across all three THP-1 cell types, indicating that only minimal levels of apoptosis are occurring in these infection conditions (**Figure 5B**).

After ruling out apoptosis as a mechanism regulated by FAK during Mtb infection, we next used the lactate dehydrogenase (LDH) assay to determine whether FAK regulates a necrotic form of cell death. The LDH assay is a well-established method of detecting necrotic cell death, since it measures cellular plasma membrane damage by the release of LDH enzyme into the culture medium. Inhibition of FAK significantly increased LDH release in Mtb-infected macrophages over a time-course of 6 days (**Figure 5C**), whereas FAK overexpressing macrophages showed decreased levels of LDH in culture supernatants compared to control macrophages (**Figure 5C**). The effect of FAK inhibition on LDH release was also confirmed in RAW 264.7 macrophages (**Figure S3**). Taken together, these findings support our hypothesis that FAK regulates a form of necrotic cell death during Mtb infection.

The two most common modes of necrotic cell death are necroptosis and pyroptosis, both of which have been implicated in TB pathogenesis (18). Our data supports that one of these pathways could be regulated by FAK given the dysregulation in TNF-α and IL-1β levels when FAK expression is modulated. Interestingly, induction of pyroptosis by activation of the inflammasome using LPS and nigericin (75) did not trigger differences in IL-1β production by THP-1 or THP-FAKi macrophages (**Figure 5D**). Surprisingly, FAK^+^ macrophages had increased sensitivity to chemical induction of pyroptosis, as indicated by increased production of IL-1β (**Figure 5D**). Together, these results indicate that inhibition of FAK does not affect inflammasome activation, and that FAK may instead promote pyroptosis when overexpressed to high levels.

To determine whether FAK instead regulates a necroptotic form of cell death during Mtb infection, we used a panel of specific inhibitors that target receptor activating protein kinase-1, -3 (RIPK1, RIPK3) and mixed lineage kinase domain-like protein (MLKL), which together form the signaling cascade and complex that leads to necroptotic cell death (76, 77). Consistent with our previous cell death assays, inhibition of FAK significantly increased cell death of Mtb-infected macrophages as indicated by reduced metabolic activity (**Figures 5E–G** and **S4**). In contrast, Mtb-infected THP-FAK^+^ macrophages showed increased metabolic activity, and therefore viability, compared to control cells (**Figures 5E–G** and **S4**). Importantly, treatment with necrostatin-1 (nec-1) (78) (**Figure S4**) or GSK-872 (79) (**Figure 5F**), inhibitors of RIPK1 and RIPK3, respectively, reversed the effects of FAK inhibition and restored cell viability to levels comparable to control macrophages. Necrostatin-1 activity has been associated with inhibition of other targets such as human IDO and T cell receptor signalling. To rule out any off-target effect with Nec-1, we repeated the experiment with a more specific inhibitor of RIPK1, necrostatin-1s (80). Treatment with nec-1s produced consistent results with that of nec-1 and showed that inhibition of RIPK1 can restore cell health of FAK inhibited macrophages during Mtb infection (**Figure 5E**). Given that MLKL is the terminal protein in the induction of necroptotic cell death, we examined whether inhibition of MLKL would also be able to restore cell viability during Mtb infection of FAKi macrophages. We demonstrate that inhibition of MLKL with necrosulfonamide (NSA) (77), resulted in a partial but significant restoration in the cell viability of FAKi macrophages infected with Mtb (**Figure 5G**). Collectively, our data suggest that FAK controls a RIPK1-RIPK3-MLKL-dependent signaling axis to regulate cell death during Mtb infection, and that RIPK1 may play a larger role in the mechanism compared to RIPK3 or MLKL.

### FAK Expression Restricts the Intracellular Survival of Mtb

**Figure 6:**
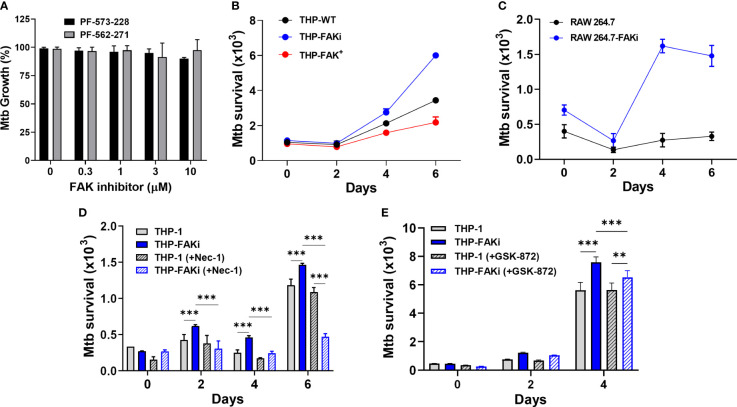
Expression of FAK controls Mtb survival.** (A)** Mtb was treated with the indicated concentrations of FAK inhibitors (in the absence of macrophages), PF-573-228 and PF-562-271, for 5 days, after which Mtb growth was assessed using the resazurin viability assay. Relative viability was normalized to 100% using untreated controls. **(B)** THP-1, THP-FAKi, and THP-FAK^+^ macrophages, or **(C)** RAW 264.7 and RAW 264.7-FAKi macrophages were infected with Mtb-luciferase at an MOI of 10 for 6 days. Infected macrophages were lysed at the indicated time points post-infection and the resultant luminescence signal (RLU) was measured as a proxy for the relative number of viable of Mtb. **(D, E)** THP-1 macrophages were mock treated or pre-treated with **(D)** 30 µM necrostatin-1 or **(E)** 5 µM GSK-872 for 24 h, and subsequently infected as in **(B)**. Infected macrophages were lysed at the indicated time points post-infection, and the resultant luminescence signal (RLU) was measured as a proxy for the relative number of viable of Mtb. Error bars represent the mean ± SD of three independent biological replicates. **p < 0.01, ***p < 0.001.

The induction of necrotic-like modes of cell death results in enhanced bacterial replication and disease progression (40). Since our results indicate that FAK has host-protective functions in the anti-Mtb response by restricting macrophage necroptosis, we sought to determine whether FAK is important in limiting Mtb burden in macrophages. Prior to assessing the effect of modulating FAK on intracellular Mtb burden, we showed that the FAK inhibitors, PF-573-228 and PF-562-271 (**Figure 6A**), do not affect Mtb viability or replication, which alleviates any concerns that the inhibitor itself can directly influence Mtb survival.

An Mtb reporter strain expressing firefly luciferase was used to perform luminescence-based infection assays to address whether FAK is required to control the intracellular survival of Mtb. Multiple studies from our lab and others support that the luciferase expression systems in Mycobacteria are strongly correlative with traditional colony forming unit (CFU) plating assays, and that luminescence (measured in Relative Light Units, RLU) maintains a linear relationship with CFU over several orders of magnitude, including within infected macrophages (60, 81, 82). THP-1 macrophages were infected with luciferase-expressing Mtb for 6 days, and the intracellular bacterial burden was measured at the indicated time points. Inhibition of FAK resulted in uncontrolled replication of Mtb (**Figure 6B**), whereas overexpression of FAK reduced the bacillary load in a time-dependent manner (**Figure 6B**) compared to THP-1 macrophages. Consistent with comparable cell death data between murine macrophages and THP-1 cells, a similar trend was observed in RAW 264.7 cells, wherein FAK inhibition resulted in increased Mtb replication (**Figure 6C**).

Importantly, treatment of macrophages with nec-1 (**Figure 6D**), significantly decreased the Mtb burden in FAK inhibited macrophages. A similar but smaller effect was observed with GSK-872 (**Figure 6E**). The ability of these inhibitors to prevent necroptotic cell death (**Figures 5E–G**), thereby resulting in decreased Mtb burden, suggests that the function of FAK in host cell necroptosis is responsible for the uncontrolled Mtb replication observed in **Figures 6B, C**. Taken together, these data suggest that FAK expression blocks necroptotic cell death of macrophages to restrict Mtb growth and survival.

### FAK Expression Promotes ROS Production to Control Mtb Infection

**Figure 7:**
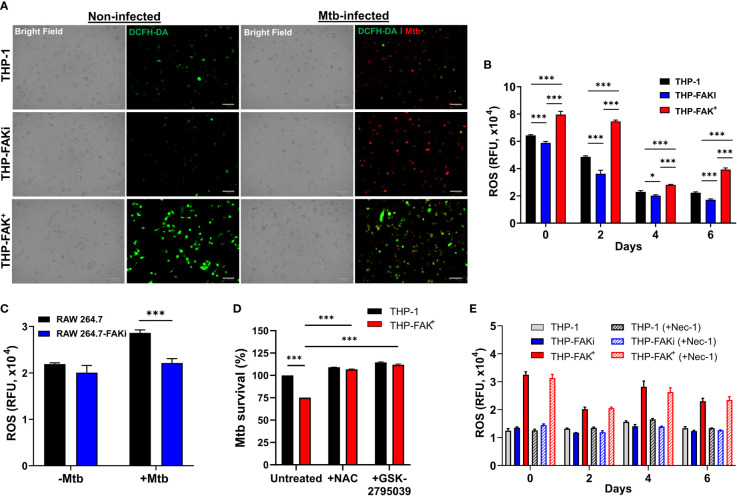
Expression of FAK induces production of ROS to control Mtb infection.** (A)** THP-1, THP-FAKi, and THP-FAK^+^ macrophages were infected with Mtb-tdTomato (red signal) for 6 days, after which cells were stained with DCFH-DA (5 μM, green signal) for 30 minutes at 37°C. Representative Bright Field and fluorescence images of uninfected and Mtb-infected (red) macrophages (green, ROS), are shown using a 20x objective. Scale bar, 100 μm. **(B)** THP-1 macrophages were infected and stained with DCFH-DA as in **(A)** and the amount of ROS production was quantified at the indicated time points post-infection using a fluorescence plate reader at 485 nm/535 nm. **(C)** RAW 264.7 macrophages were infected and stained as in **(A)** and the amount of ROS production was quantified at day 6 post-infection. **(D)** THP-1 and THP-FAK^+^ macrophage were pre-treated with 10 mM N-acetyl-cysteine (NAC) or 25 µM GSK2795039 for 24 h and then infected with Mtb-luciferase at an MOI of 10. Infected macrophages were lysed at day 6 post-infection and the resultant luminescence signal was measured as a proxy for the relative number of viable of Mtb. RLU signals were normalised to mock treated THP-1 cells as 100% and depicted as % Mtb survival. **(E)** THP-1, THP-FAKi, and THP-FAK^+^ macrophages were mock treated or pre-treated with 30 µM necrostatin-1 for 24 h, after which cells were infected with Mtb at MOI of 10. At indicated time points post-infection, infected macrophages were stained with DCFH-DA as in **(A)** and fluorescence signals for ROS production was measured. Error bars represent the mean ± SD of three independent biological replicates. *p < 0.05, ***p < 0.001.

Since lack of cell death is not by itself a direct antibacterial response, we sought to identify alternative mechanisms/pathways that would explain the increased ability of FAK overexpression macrophages to restrict intracellular Mtb survival. Host cells use antibacterial mechanisms such as increased induction of ROS and reactive nitrogen intermediates (RNI) to control Mtb growth and survival (6, 26). Interestingly, studies have reported that FAK plays a crucial role in NADPH oxidase-dependent ROS production to reduce the survival of *Escherichia. coli* in neutrophils (83). To determine whether FAK regulates ROS production to control Mtb survival, THP-1, THP-FAKi and THP-FAK^+^ macrophages were infected with Mtb and production of ROS was measured using the DCFH-DA probe at the indicated time points. Overexpression of FAK increased production of ROS in Mtb-infected macrophages compared to wild type macrophages, whereas inhibition of FAK dampened ROS production as demonstrated by the intensity of ROS staining visualized with immunofluorescence microscopy (**Figure 7A**) and quantified with multi-mode plate reader (**Figure 7B**). The same observation was confirmed in RAW 264.7 macrophages as inhibition of FAK decreased ROS production during Mtb infection (**Figure 7C**). This result suggests that production of ROS may be another antibacterial pathway regulated by FAK to control Mtb survival. To evaluate whether the production of ROS is directly responsible for improved control of Mtb by FAK^+^ macrophages, we evaluated Mtb survival in THP-1 and THP-FAK^+^ macrophages in the presence of antioxidants. Treatment of cells with N-acetyl-cysteine (NAC) restored the intracellular survival of Mtb in FAK overexpressing macrophages to levels of wild-type THP-1 macrophages (**Figure 7D**). To account for the possibility that this restoration could be the result of anti-Mtb properties of NAC (84), we confirmed our finding with another inhibitor of ROS production, GSK2795039 (85), which inhibits NOX2. Treatment of macrophages with GSK2795039 also restored Mtb survival in FAK^+^ macrophages (**Figure 7D**). Interestingly, the impact of FAK on ROS production is independent of cell death, since we observed no significant differences in ROS production between necrostatin-1-treated and untreated macrophages (**Figure 7E**). As such, there appears to be no direct association between ROS production and cell death mediated by FAK. Collectively, our data establishes FAK as an important host-protective kinase that is exploited by Mtb to induce necrotic cell death and suppress ROS production, thereby promoting bacterial survival.

## Discussion

In this study, we demonstrate that Mtb infection suppresses FAK expression in macrophages in a time-dependent manner (**Figure 1**). Since a canonical function of FAK is to regulate the survival of adherent cells (86, 87), downregulation of its expression during Mtb infection could be a strategy employed by the bacteria to induce macrophage cell death and disable the antibacterial response. With this observation, we had anticipated that FAK may have host-protective functions to aid in Mtb clearance. However, this phenomenon was not initially supported by our cytokine profile data, since FAK enhanced the production of anti-inflammatory IL-10 (**Figure 3C**) while inhibition of FAK promoted the production of proinflammatory cytokines TNF-α (**Figure 3A**) and IL-1β (**Figure 3B**) in Mtb-infected macrophages. These results conflict with findings from studies conducted in vascular (88) and aortic inflammation (89), where FAK mediates the increased production of proinflammatory cytokines, TNF-α and IL-1β, to control macrophage behaviour. However, the context of vascular and aortic inflammation is different than that of bacterial infections, where the role of FAK in inflammation has not been explored. Consistent with our data, FAK plays an immunomodulatory role in mediating COX2 mechanoresponse to suppress TNF-α production in bone marrow mesenchymal stromal cells (90). IL-10 is generally associated with increased Mtb burden (91–94). However, IL-10 also provides a host-protective role in restraining hyperinflammation to prevent excessive tissue damage during specific phases of TB disease (93, 95). In contrast, TNF-α and IL-1β are generally considered to be essential for the control of TB infection; however, excess levels can be detrimental to the host by causing macrophage cell death and subsequent hyperinflammation (26). Taken together, our findings suggest that FAK is associated with reduced inflammation during TB infection, whereas inhibition of FAK increases inflammation. While it may seem counter-intuitive for Mtb to trigger increased inflammation *via* downregulation of FAK, a probable explanation is that the increased inflammation could be a result of necrotic cell death that is known to be induced by Mtb (96, 97).

Indeed, our data demonstrate that FAK is essential for controlling cell death during Mtb infection as measured by multiple parameters of macrophage health, including adherence, metabolic activity, and plasma membrane integrity (**Figures 4A–E**). Our observation that inhibition of FAK results in increased cell death of Mtb-infected macrophages was expected given the established role of FAK in the dynamic regulation of integrin-based adhesion and the actin cytoskeleton, which is crucial for the control of cell survival (98). FAK also modulates the life span of other cell types such as neutrophils, where deletion of FAK impairs fibronectin- and ICAM-1-based adhesion, leading to spontaneous cell death (83). As such, macrophages with reduced FAK expression during Mtb infection may experience similar disruptions in adhesion, thus triggering the activation of cell death programs. In addition, our observation that overexpression of FAK can inhibit cell death during Mtb infection is consistent with studies in which increased expression of FAK blocks cell death to increase survival and migration of cancer (99) and endothelial cells (100).

Given that different modes of cell death have different effects on host immunity against bacterial infections, particularly in TB wherein both acute and chronic phases of infection exist, we wanted to address the specific mode of death regulated by FAK during Mtb infection. We had originally anticipated that FAK may regulate apoptosis in macrophages during Mtb infection, since FAK is known to modulate apoptosis in a wide range of cancers (49, 51). Unexpectedly, our data show that apoptosis is not the mode of cell death regulated by FAK (**Figures 5A, B**). Other reports have highlighted that excess production of TNF-α and IL-1β trigger necrosis of Mtb-infected macrophages through the production of mitochondrial ROS and the participation of cyclophilin D, a component of the mitochondrial permeability transition pore (41, 101). Indeed, using chemical inhibitors of necroptosis, our data suggest a new role for FAK in the regulation of necroptosis during Mtb infection (**Figure 5**), which is consistent with the increased production of TNF-α and IL-1β (**Figures 3A, B**), increased plasma membrane damage (**Figure 4E**), and increased release of LDH (**Figure 5C** and **Figure S3**). Considering that Mtb is known to induce necrotic forms of cell death while suppressing apoptosis (19, 25), our data indicate that Mtb exploits the downregulation of FAK to induce necroptosis during infection. Interestingly, our data indicate that RIPK1 plays a more important role than RIPK3 and MLKL in FAK-mediated cell death during Mtb infection. While inhibition of RIPK3 or MLKL only partially restored cell death or Mtb survival in FAKi treated macrophages, inhibition of RIPK1 almost completely restored the phenotype to that of wild-type cells (**Figures 5E–G** and **6D–E**). While RIPK3 and MLKL are almost exclusively associated with necroptotic cell death, RIPK1 is known to play roles in not only necroptosis but also apoptosis and pyroptosis (102, 103). Our data show that FAK does not contribute to apoptosis or pyroptosis; however, our experiments in the context of pyroptosis relied on chemical activation of inflammasome, which may be entirely different when compared to bacterial infection. As such, our results do not completely exclude the role of FAK in a pyroptosis during Mtb infection, nor other necrotic forms of cell death that are dependent on RIPK1.

The type of cell death largely influences TB disease progression and the outcome of infection since macrophages provide a protective intracellular niche for the bacteria to persist and replicate. Host cell apoptosis is associated with a significant decrease in intracellular Mtb growth (30), whereas necrotic cell death is detrimental to the control of Mtb burden as increased bacterial burden eventually leads to spread of the bacteria to the extracellular milieu (40, 96). We observed that FAK inhibition in human macrophages leads to uncontrolled replication of Mtb, whereas overexpression of FAK limits the intracellular survival of Mtb in macrophages (**Figures 6B, C**). These data support a role for FAK as an important host signaling protein during Mtb infection that blocks necrotic cell death and restricts the survival of Mtb. In addition, our data show an antibacterial effect associated with increased FAK expression, which is consistent with a previous report in a mouse peritonitis model where FAK expression increased the capability of neutrophils to kill *Escherichia coli* (83). On the other hand, our work also contradicts findings where FAK uses a plethora of cellular signalling networks and mechanisms to favour the attachment, entry and replication of extracellular microorganisms ranging from bacteria (54, 55), viruses (104, 105) and fungi (106) in host target cells. These disparate findings on the role of FAK during infection by various pathogens suggest that this kinase participates in both host-protective and host-detrimental functions, likely through differing cellular mechanisms in response to different pathogens. Based on our results in the context of Mtb infection, we speculate that increased FAK expression may be required to control the survival of intracellular pathogens in host macrophages.

While increased necrosis has been associated with increased Mtb survival, it remains unknown whether this is the direct result of the cell death or if other processes play a role to enhance bacterial killing. This question was compelling when considering the mechanism of how FAK overexpression results in reduced Mtb survival relative to control macrophages (**Figures 6B, C**). To eliminate Mtb during infection, host cells trigger a range of antibacterial responses, including increased induction of ROS (6), RNI (26), phagosome maturation (107), autophagy (10), and efferocytosis (108). Our data suggest a new role for FAK in the generation of ROS to control intracellular Mtb survival (**Figures 7A, B**). Inhibition of NOX2 or scavenging of ROS restored the intracellular survival of Mtb in FAK^+^ macrophages, further supporting a direct antibacterial effect of FAK-mediated ROS production (**Figure 7D**). Although ROS production is important for host resistance against Mtb, excess ROS leads to oxidative stress and concomitant necrosis. However, it is surprising how enhanced production of ROS by FAK expression did not exacerbate necrosis, indicating that tolerable levels of ROS are produced to eliminate the pathogen without damaging the host macrophage. We show that expression of FAK is required for full macrophage bacterial killing capacity, consistent with our finding that FAK inhibition disrupts bactericidal processes such as NADPH oxidase-mediated superoxide production (**Figure 7**). Importantly, this phenomenon is also observed in FAK-deficient neutrophils in the context of *Escherichia coli* infection (83), highlighting the relevance of FAK as a key regulator in ROS production. Studies have also shown that proteins of the extracellular matrix, including FAK-activated Rho GTPases, participate in ROS production through mitochondrial engagement (109). Interestingly, our work showed no link between ROS production and cell death mediated by FAK (**Figure 7E**), suggesting that FAK-mediated regulation of cell death and ROS production rely on two distinct pathways. The exact mechanisms by which FAK controls necrosis remains to be uncovered. Since nuclear FAK interacts with both p53 and the E3 ubiquitin ligase Mdm2, leading to p53 degradation and the suppression of apoptosis (110), identifying the subcellular localization of FAK during Mtb infection may provide insight into the mechanism by which FAK regulates macrophage necrosis. Ultimately, it will be important to corroborate our *in vitro* findings with *in vivo* models, as this will establish physiological relevance for the role of FAK in the host immune response to TB.

Overall, our study reveals an important role for FAK as a host-protective protein during Mtb infection that blocks necrotic cell death and induces ROS production, which are required to restrict the survival of Mtb. Importantly, we have demonstrated that pharmacological inhibition of FAK is detrimental to host macrophages during Mtb infection, whereas genetic overexpression of FAK restricts Mtb survival. As such, our findings suggest that targeting FAK using activators such as phosphatidylinositol 4,5-bisphosphate (111) could be a desirable strategy for HDT against Mtb infection. Since FAK expression decreases inflammation, activators of FAK could also prevent long-term lung pathology in TB patients. The usage of such pharmacological agents for TB HDT could have important clinical implications by improving the anti-TB immune responses to facilitate pathogen clearance.

## Data Availability Statement

The original contributions presented in the study are included in the article/**Supplementary Material**. Further inquiries can be directed to the corresponding author.

## Ethics Statement

The animal study was reviewed and approved by the Animal Care Committee of the University of Ottawa (Protocol #3132).

## Author Contributions

AA-A, AnD, and JS conceived the study, designed experiments, analyzed data, and wrote the manuscript. AA-A, AnD, AmD, SB, and RS performed experiments. AmD contributed to the original draft of the manuscript. All authors read, edited, and approved the final manuscript for submission.

## Funding

This work was supported by grants from the Canadian Institutes of Health Research (CIHR) PJT-162424, the National Sanitarium Association Scholar’s Program, the Lung Health Foundation Breathing as One - Young Investigators Research Award, and the Natural Sciences and Engineering Research Council of Canada (NSERC) RGPIN-2020-04032 to JS.

## Conflict of Interest

The authors declare that the research was conducted in the absence of any commercial or financial relationships that could be construed as a potential conflict of interest.

## Publisher’s Note

All claims expressed in this article are solely those of the authors and do not necessarily represent those of their affiliated organizations, or those of the publisher, the editors and the reviewers. Any product that may be evaluated in this article, or claim that may be made by its manufacturer, is not guaranteed or endorsed by the publisher.
