# Supplementary material for: Mycobacterium tuberculosis Exploits Focal Adhesion Kinase to Induce Necrotic Cell Death and Inhibit Reactive Oxygen Species Production
Source: Front Immunol. 2021 Oct 20;12:742370. doi: 10.3389/fimmu.2021.742370 (PMC8564185; doi:10.3389/fimmu.2021.742370)
Supplement: Supplementary file 1 [file DataSheet_1.pdf]

Supplementary Figures

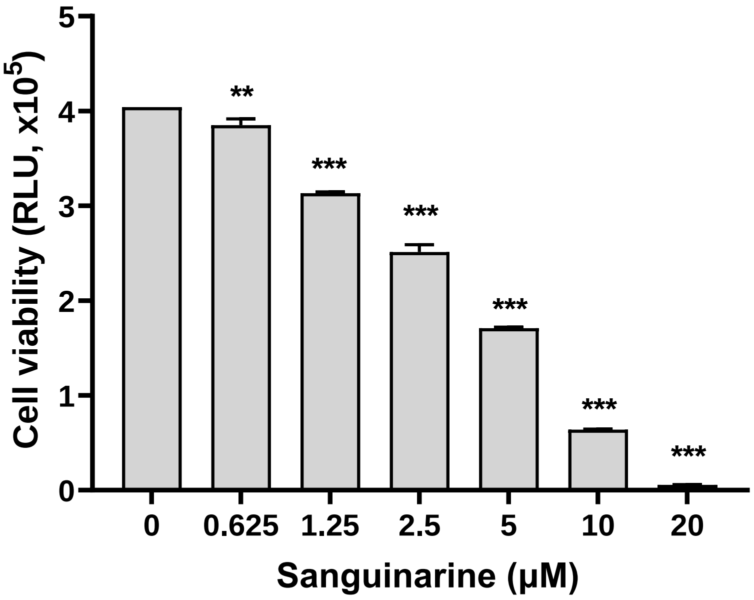

Figure S1. Use of the Cell Titer-Glo assay to measure cytotoxicity in macrophages. THP-1 macrophages were treated with increasing concentrations of sanguinarine and cell viability was quantified at 48 h post-treatment using the Cell Titre-Glo assay (measured as relative luminescence units, RLU). Data represents the mean ± SD of three independent biological replicates. **$p < 0.01$, ***$p < 0.001$.

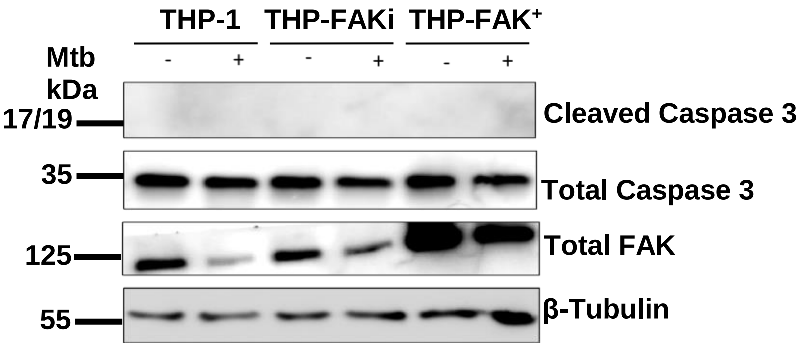

Figure S2. FAK-mediated cell death during Mtb infection is independent of caspase-3 activation. THP-1, THP-FAKi, and THP-FAK^{+} macrophages were infected with Mtb at an MOI of 10 and cell lysates were prepared 24 hours post-infection. Cleaved caspase 3, total caspase 3 and total FAK levels were analyzed by western blotting. β-Tubulin was used as loading control.

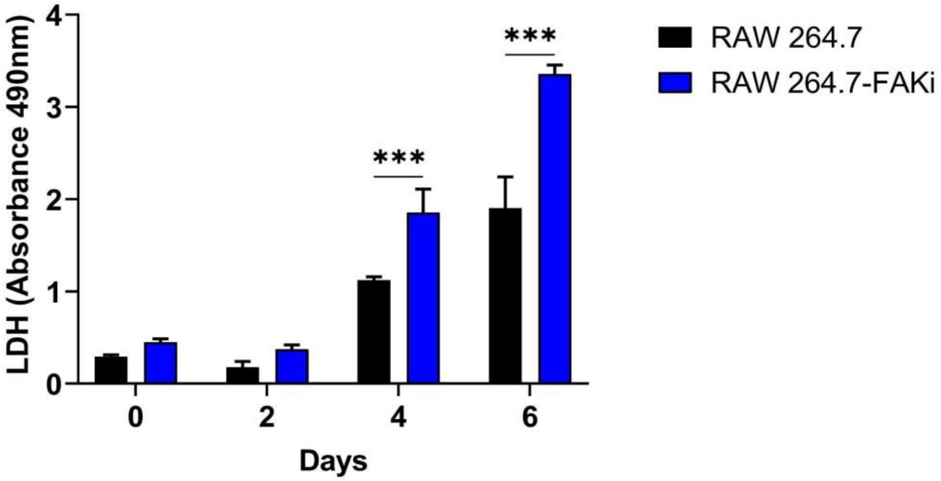

Figure S3. Inhibition of FAK induces necrotic cell death in Mtb-infected RAW 264.7 macrophages. RAW 264.7 and RAW 264.7-FAKi macrophages were infected with Mtb at an MOI of 10, and lactate dehydrogenase (LDH) released in culture supernatants were assessed using the CYQUANT LDH kit at indicated time points post-infection. Error bars represent the mean ± SD of three independent biological replicates. *** $p < 0.001$.

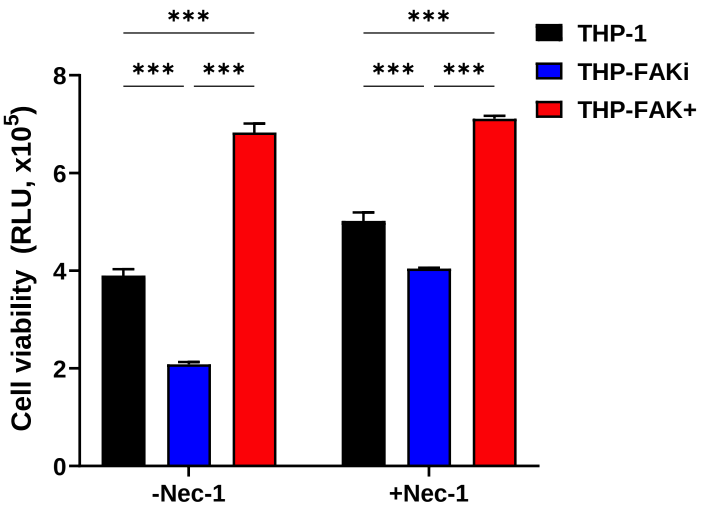

Figure S4. Inhibition of RIPK1 restores cell health in FAKi macrophages during Mtb-infection. THP-1, THP-FAKi, and THP-FAK$^+$ macrophages were mock treated or pre-treated with 30 µM necrostatin-1 for 24 hours. Cells were then infected with Mtb at an MOI of 10 for 6 days, and cell viability was assessed using Cell Titer-Glo. Error bars represent the mean ± SD of three independent biological replicates. *** $p < 0.001$.
